# Mechanism of continuous high temperature affecting growth performance, meat quality, and muscle biochemical properties of finishing pigs

**DOI:** 10.1186/s12263-019-0643-9

**Published:** 2019-07-24

**Authors:** Xianyong Ma, Li Wang, Zibiao Shi, Wei Chen, Xuefen Yang, Youjun Hu, Chuntian Zheng, Zongyong Jiang

**Affiliations:** 1 Institute of Animal Science, Guangdong Academy of Agricultural Sciences, Guangzhou, 510640 People’s Republic of China; 2 The Key Laboratory of Animal Nutrition and Feed Science (South China) of Ministry of Agriculture, Guangzhou, China; 3 State Key Laboratory of Livestock and Poultry Breeding, Guangzhou, China; 4 Guangdong Key Laboratory of Animal Breeding and Nutrition, Guangzhou, China; 5 Guangdong Engineering Technology Research Center of animal Meat quality and Safety Control and Evaluation, Guangzhou, 510640 China

**Keywords:** Growth performance, High ambient temperature, Meat quality, mRNA array, Restricted feed intake

## Abstract

**Background:**

The mechanism of high ambient temperature affecting meat quality is not clear till now. This study investigated the effect of high ambient temperature on meat quality and nutrition metabolism in finishing pigs.

**Methods:**

All pigs received the same corn-soybean meal diet. A total of 24 Landrace × Large White pigs (60 kg BW, all were female) were assigned to three groups: 22AL (fed ad libitum at 22 °C), 35AL (ad libitum fed at 35 °C), and 22PF (at 22 °C, but fed the amount consumed by pigs raised at 35 °C) and the experiment lasted for 30 days.

**Results:**

Feed intake, weight gain, and intramuscular fat (IMF) content of pigs were reduced, both directly by high temperature and indirectly through reduced feed intake. Transcriptome analysis of longissimus dorsi (LM) showed that downregulated genes caused by feed restriction were mainly involved in muscle development and energy metabolism; and upregulated genes were mainly involved in response to nutrient metabolism or extracellular stimulus. Apart from the direct effects of feed restriction, high temperature negatively affected the muscle structure and development, energy, or catabolic metabolism, and upregulated genes were mainly involved in DNA or protein damage or recombination, cell cycle process or biogenesis, stress response, or immune response.

**Conclusion:**

Both high temperature and reduced feed intake affected growth performance and meat quality. Apart from the effects of reducing feed intake, high temperature per se negatively downregulated cell cycle and upregulated heat stress response. High temperature also decreased the energy or catabolic metabolism level through PPAR signaling pathway.

**Electronic supplementary material:**

The online version of this article (10.1186/s12263-019-0643-9) contains supplementary material, which is available to authorized users.

## Introduction

Continuous high temperature, especially in summer in tropical or subtropical countries, is an unfavorable factor in swine production. Persistent exposure to high temperature decreases feed intake [1], growth performance [2], and meat quality [3, 4]. For example, high temperature reduced intramuscular fat (IMF) deposition [5, 6] and changed the pH value of the meat [3, 7]. These alterations were traditionally believed to result from the decreased feed intake, but more recent studies have shown that heat stress per se also reduced metabolic rates and altered post-absorptive metabolism, regardless of decreased feed intake [8, 9]. Heat stress also changed expression of some genes related to oxidative metabolism, through adaptive physiological mechanisms, to reduce thermogenesis [7, 10]. Although inferior meat quality induced by heat stress has been intensively studied, the molecular mechanisms underlying the pathophysiological changes remain to be defined. As heat stress does decrease feed intake, it remains unclear what changes are dependent or independent low nutrient availability. Gene expression profiles of longissimus muscle (LM) have been used here to further examine how heat stress affects meat quality and the extent to which it is dependent on reduced feed intake.

## Materials and methods

### Animals and diets

A total of 24 Landrace × Large White pigs (60 kg BW) were assigned randomly to three groups with eight pigs per group. Pigs were housed individually in wire cages (139 × 67 × 115 cm) in one of three temperature-controlled rooms at the Institute of Animal Science, Guangdong Academy of Agricultural Sciences. After adaption for 1 week, pigs were treated as follows: a control group of pigs had ad libitum access to feed at 22 °C (RT) (22AL); the heat-stressed group had ad libitum access to feed at 35 °C (35AL); and pair-fed pigs at 22 °C (22PF) were fed the amount consumed by pigs raised at 35 °C. All pigs were fed twice daily with a typical corn-soybean meal-based diet for finishing pigs (the diet formula is available as Additional file 1: Table S1). The temperature in one room was increased from 22 to 35 °C within approximately 2 h and then remained at 35 °C for the 30-d experimental period; other rooms were maintained at 22 °C. Water was available ad libitum for all pigs.

### Feeding, slaughter procedure, and sample collection

All aspects of the experiment including transport and slaughtering procedures were carried out in accordance with the Chinese guidelines for the use of experimental animals and animal welfare [11] and approved by the Animal Experimental Committee of the Institute of Animal Science, Guangdong Academy of Agricultural Sciences. Pigs were weighed at the beginning and feed intakes were recorded to determine average daily gain (ADG), average daily feed intake (ADFI), and feed to gain ratio (F:G). At the end of the experiment, all pigs were fasted for 14 h, and then slaughtered; the whole LM samples were taken immediately after slaughtered for testing meat quality and parts samples at the last thoracic vertebra were collected and stored at − 80 °C for subsequent analyses.

### Meat quality measurements

The pH of muscle samples was measured at 45 min, 24 h, and 48 h postmortem using a pH meter (HI 8242C, Beijing Hanna Instruments Science & Technology, Beijing, China). Meat color CIE LAB values (L*, lightness; *a*, redness; *b*, yellowness) were determined on the transverse surface of the LM after it was cut and let to bloom for 45 min at the same times postmortem using a colorimeter (CR-410, Minolta, Suita-shi, Osaka, Japan), as described by Mason et al. [12]. Shear force was measured using an Instron Universal Mechanical Machine (Instron model 4411; Instron, Canton, MA, USA) and drip loss was measured by weight loss over 24 h at 4 °C in a plastic bag, also as described by Mason et al. [12]. The IMF content was measured by petroleum ether extraction of powdered, lyophilized muscle using the Soxtec 2055 fat extraction system (Foss Tecator AB, Höganäs, Sweden), according to the Association of Official Analytical Chemists method [13].

### RNA extraction and target labeling

Total RNA was isolated from LM using the TRIzol reagent (Invitrogen, Carlsbad, CA, USA) and purified using a QIAGEN RNeasy® Mini Kit (QIAGEN, Chatsworth, CA, USA) according the manufacturer’s instructions. The RNA quality was checked with a spectrophotometer (ND-1000, Nano-Drop Technologies, Wilmington, DE, USA). Each RNA sample was annealed with a primer containing a poly-dT and a T7 polymerase promoter. Reverse transcriptase produced primary and secondary cDNA strands. T7 RNA polymerase was then used to create cRNA from the double-stranded cDNA by incorporating cyanine-3-labeled cytidine 3-CTP according to the labeling kit recommendations (Agilent Technologies, Santa Clara, CA, USA). The quality of the labeled cRNA was again verified.

### Hybridization, scanning, and feature extraction

A total of 24 pigs were used and four cRNA pools of two pigs per treatment was hybridized to 12 microarrays (4 × 3 treatments) using a Gene Expression Hybridization Kit (Agilent) at 60 °C for 17 h using whole pig genome arrays (Pig 4x44K Gene Expression Microarrays v2, Agilent). The arrays were washed, stabilized, and dehydrated, as recommended, then examined on a G2565BA microarray scanner (Agilent) and the data were compiled using feature extraction software (FE).

### Data analysis of the mRNA microarrays

Array normalizations and error detection were carried out using Silicon Genetics’ GeneSpring GX v11.5.1 (Agilent) via the enhanced FE import preprocessor, then was normalized using the supplied algorithms. A final quality control filter was applied to eliminate transcripts with excessive biological variability then GeneSpring was used to reveal genes significantly differing in expression among the 3 treatments. Differentially expressed genes with statistical significance between pairs of treatments were those passing Volcano Plot filtering (Fold Change ≥ 2.0, *P* value ≤ 0.05).

### Real-time PCR analysis

Total RNA, extracted as described above, was used to prepare cDNA (four pools of two pigs per treatment) with PrimeScript RT reagent kits (Takara, Ostu, Japan) according to the manufacturer’s instruction. Real-time PCR was carried out on a CFX connect Real-Time System (Bio-Rad, Hercules, CA) using the iTaq Universal SYBR Green SuperMix (Bio-Rad Laboratories) with gene-specific primers. The primer sequences (listed in Table 4) were designed using Primer Express 5. The PCR protocol was as follows: denaturation at 95 °C for 30 s, followed by 40 cycles of 95 °C for 20 s and 60 °C for 20 s, then 72 °C for 30 s. The relative abundance of transcripts was expressed as 2^-∆∆Ct^, where the Ct (threshold cycle) value for each reaction was used to calculate gene expression, and then further normalized to abundance in the 22AL control animals, given a value of 1.

### Western blotting

Pools (four pools of two pigs per treatment) of muscle proteins (40 μg, one for each treatment) were separated by SDS-PAGE and proteins were electro-transferred to PVDF membranes at 250 mA for 90 min, as described by Ma et al. [14]. Membranes were blocked overnight at 4 °C with 5% non-fat milk in TBST (10 mmol/L Tris-HCl, pH 8.0, 150 mmol/L NaCl, 0.1% Tween 20). All primary antibodies were raised in rabbits and were suitable for detecting specific porcine proteins: DECR1(sc-393473, Santa Cruz Biotechnology, Dallas, TX), FABP3 (orb156814, Biorbyt, Berkeley, CA), HSPA1L (bs-18079R, Bioss, Columbia, TN), LPIN1 (sc-50050, Santa Cruz), PEKM (sc-31712, Santa Cruz), TNNI1 (orb106871, Biorbyt), TNNT3 (BWB-MS201C, Genway Biotech, San Diego, CA), PPARGC-1 (bs-7535R, Bioss), MSTN (ab98337, Abcam), FASN (ab128856, Abcam), PCK1 (ab87340, Abcam), and GAPDH (sc-20357, Santa Cruz). Each was used at 1:250 diluted in blocking buffer for 2 h at room temperature. Membranes were washed three times, each for 10 min, with 10 mL TBST then incubated for 1 h in blocking buffer containing horseradish peroxidase-labeled (HRP) anti-rabbit secondary antibody (ABCAM Biotechnology, Cambridge, UK) diluted 1:5000. After three 10-min washes, immunoreactive proteins were visualized using a chemiluminescent HRP substrate (Millipore, Billerica, MA) and a Versa Doc imaging system (Bio-Rad). The band densities were calculated by Quantity One software (Bio-Rad) and compared to the density of GADPH. The Western blot analyses were performed in triplicate and data were analyzed with IMAGE J 1.49 software (NIH, Bethesda, MD).

### Statistical analysis

The effect of treatment was examined by one-way ANOVA and, where appropriate, means were compared using Fisher’s least significant difference (LSD) post-hoc tests (SPSS V22). Differences were considered to be significant at *P* <  0.05. Microarray analyses were conducted using GeneSpring GX v11.5.1, via the enhanced FE import preprocessor, then normalized using the supplied algorithms. Significant GO ID pathways were selected by adjusted *P* value < 0.05.

## Results

### Growth performance and meat quality

The performance and meat quality data are summarized in Table 1. The final BW, ADFI, and ADG of the control pigs (22AL) were much higher than those in the heat-stressed (35AL) or pair-fed pigs (22PF) (*P* < 0.05), but the backfat thickness of the first rib in the control was lower than that in the heat-stressed or pair-fed pigs (*P* < 0.05), the latter having the lowest F:G ratio. There were no differences on loin eye area and leaf fat weight among three groups (*P* > 0.05). The pH value of LM in 35AL pigs was higher than that in the PF pigs at 45 min postmortem (*P* < 0.05), was higher than that of other two treatments at 24 h (*P* < 0.05), and exceeded that of the 22AL pigs at 48 h (*P* < 0.05). There were no differences in drip loss at 24 h or 48 h postmortem, nor in meat color (*a*, *b*, and L* value) except for L* at 45-min postmortem being lowest in the 22AL controls(*P* < 0.05). Both high temperature and feed restriction decreased IMF content in LM (*P* < 0.05). The shear force value in 22PF pigs was greatest in 22PF pigs (*P* < 0.05), without any difference between 35AL and 22AL pigs.

**Table 1:** Effect of high temperature and feed restriction on growth performance and meat quality of finishing pigs

Variable		Treatment			S.E.M	*P* value
		22AL	22PF	35AL		
		(22 °C, ad lib)	(22 °C, pair-fed)	(35 °C, ad lib)		
Initial BW, kg		77.75	77.62	77.80	0.416	0.630
Final BW, kg		103.85	86.02	87.70	10.12	< 0.001
ADG, kg/day		0.87^a^	0.28^b^	0.33^b^	0.061	< 0.001
ADFI, kg/day		3.02^a^	1.68^b^	1.51^b^	0.146	< 0.001
G:F		0.29^b^	0.17^a^	0.22^b^	0.015	0.001
Backfat first rib, mm Thickness tenth rib, mm Last rib, mm		33.67^a^	39.09^b^	42.77^b^	0.862	0.043
		21.48	22.04	22.27	0.974	0.715
		14.05	17.63	18.85	0.315	0.057
Loin eye area, mm^2^		4766	4431	4355	0.507	0.431
Leaf fat weight, kg		0.525	0.534	0.586	0.442	0.373
pH	45 min	6.10^b^	6.15^ab^	6.20^a^	0.04	0.113
	24 h	5.48^b^	5.48^b^	5.57^a^	0.014	0.002
	48 h	5.52^b^	5.55^ab^	5.64^a^	0.022	0.053
Drip loss,%	24 h	2	2	2.05	0.095	0.977
	48 h	2.61	2.48	2.59	0.101	0.872
L*	45 min	45.25^b^	46.58^a^	47.45^a^	0.303	< 0.001
	24 h	57.87	58.19	58.11	0.415	0.926
	48 h	57.75	58.18	58.87	0.386	0.082
*a*	45 min	15.84	15.58	15.81	0.203	0.864
	24 h	15.39	14.67	14.42	0.147	0.511
	48 h	15.12	15.55	15.06	0.210	0.612
*b*	45 min	2.31	2.61	2.59	0.112	0.503
	24 h	3.98	3.4	3.62	0.142	0.269
	48 h	3.11	3.16	3.23	0.167	0.96
IMF,%		4.77^a^	3.03^b^	2.89^b^	0.351	0.05
Shear force, *N*		41.51^b^	49.01^a^	43.15^b^	1.127	0.038
Glycogen content, mg/g		2.15^a^	1.38^b^	1.19^b^	0.681	0.046

Data are means with s.e.m., *n* = 8

a, b, Within a row, means with a common superscript do not differ (*P* > 0.05)

*ADG* average daily gain, *ADFI* average daily feed intake, *F*:*G*, feed gain ratio, *IMF* intramuscular fat, L* lightness, *a* redness, *b* yellowness

### mRNA expression profiling and bioinformatics analysis

**Fig. 1:**
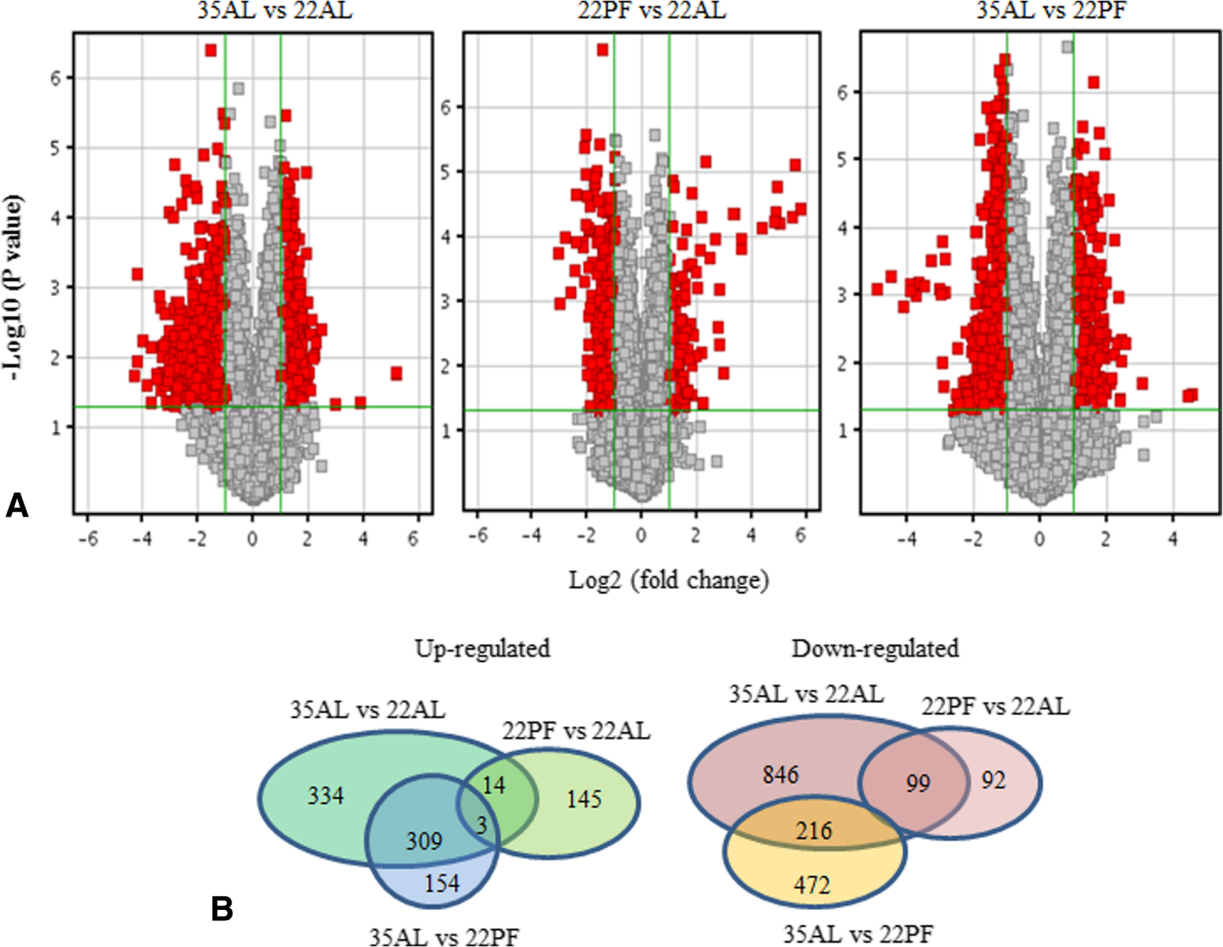
Differentially expressed genes in high temperature (35AL) vs. control (22AL), pair-fed (22PF) pigs vs. controls (22AL), high temperature (35AL) vs. pair-fed (22PF) pigs. **a** Volcano plot, red part stands for *P* value smaller than 0.05 and an average fold change (FC) of at least 2 in either direction. **b** Number of differentially expressed genes between treatments

More than 22,100 pig probes were detectable and the 22AL, 22PF, and 35AL treatments were compared and significant changes (≥ 2.0-fold, *P* ≤ 0.05) are described here. Compared with the 22AL controls, 660 genes were upregulated and 1159 genes downregulated in the 35AL pigs while 162 genes were upregulated and 191 genes downregulated in the pair-fed, 22PF pigs. To assess effects of high temperature distinct from the reduced level of intake, the comparison between 22PF and 22AL pigs identified 466 upregulated and 688 downregulated genes (Fig. 1). From these differentially expressed genes, parts of representative genes related to lipid metabolism, glucose metabolism, muscle structure development, and cellular response to stress were selected and listed in Table 2. Compared with the control, most of the lipid metabolic process related genes in 35AL group were downregulated such as ACSL1(acyl-CoA synthetase long-chain family member 1), ITGAV (integrin, alpha V), FABP3 (fatty acid binding protein 3), DLD (dihydrolipoamide dehydrogenase), CPT1B (carnitine palmitoyltransferase 1B), LPL (lipoprotein lipase), SCD (stearoyl-CoA desaturase), DECR1 (2,4-dienoyl CoA reductase 1, mitochondrial), ACADL (acyl-CoA dehydrogenase, long chain), ACADM (acyl-CoA dehydrogenase), ACOX1 (acyl-CoA oxidase 1), CYP2E1 (cytochrome P450, family 2, subfamily E, polypeptide 1), ATP5B (ATPase 5B), CPT1A (carnitine palmitoyltransferase 1A), LPINI (lipin 1), while FASN (fatty acid synthase), CYP2E1, and PPARGC-1 (peroxisome proliferator activated receptor gamma, coactivator 1 alpha) were upregulated; DECR1, CPT1A, and PPARGC-1 in 22PF group were downregulated. Compared with 22PF group, ITGAV, DLD, DECR1, ACADM, ATP5B, and APOC3 (apolipoprotein C-III) were downregulated. Glucose metabolic process related genes (UGP2(UDP-glucose pyrophosphorylase 2), GPI (glucose-6-phosphate isomerase), GPD1 (glycerol-3-phosphate dehydrogenase 1), PFKM (phosphofructokinase), TPI1 (triosephosphate isomerase 1), PKM2 (Pyruvate kinase isozyme type M2), PGM1 (Phosphoglucomutase 1)) expression in 35 AL group were lower than those in 22AL and 22PF group. Compared with control, PCK1 was upregulated in 35AL and 22PF group. All the genes related with muscle structure development listed in Table 2 were downregulated in 35AL group and most of them were downregulated in 22PF group compared with the control. Cellular response to stress-related genes such as GTF2H4 (general transcription factor IIH subunit 4), ERCC1 (excision repair cross-complementing rodent repair complementation group 1), HSPA1L (heat shock 70 kDa protein 1-like), MLH1 (mutL homolog 1), GADD45A (growth arrest and DNA- damage-inducible, alpha), CDKN1A (cyclin-dependent kinase inhibitor 1A), MAPK14 (mitogen-activated protein kinase 14), and TICAM2 (toll-like receptor adaptor molecule 2) were upregulated in 35AL group and some of them were upregulated in 22PF group compared with the control. Cellular response to hormone stimulus related genes such as NR3C1, GHR, ACVR1, ADRB2, IFNGR2, FOLR2, and ADIPOR1 in 35AL group was downregulated and some of them in 22PF group was downregulated compared with the control.

**Table 2:** Differentially expressed genes related to energy metabolism, muscle structure, and stress response

Gene^a^	Description	Fold change^b^		
		35AL vs. 22AL	22PF vs. 22AL	35AL vs. 22PF
Lipid metabolic process				
*ACSL1*	Acyl-CoA synthetase long-chain family member 1	0.22		0.45
*ITGAV*	Integrin, alpha V	0.25		
*CD36*	CD36 molecule	0.28		
***FABP3***	Fatty acid binding protein 3	0.42	0.46	
*DLD*	*Sus scrofa* dihydrolipoamide dehydrogenase	0.22		0.29
*CPT1B*	Carnitine palmitoyltransferase 1B	0.48		
***LPL***	Lipoprotein lipase	0.35		
*SCD*	Stearoyl-CoA desaturase	0.18		
***DECR1***	2,4-Dienoyl CoA reductase 1, mitochondria	0.24	0.50	0.50
*ACADL*	Acyl-CoA dehydrogenase, long chain	0.47	0.39	
*ACADM*	Acyl-CoA dehydrogenase	0.39		0.46
*ACOX1*	Acyl-CoA oxidase 1, palmitoyl	0.44		
*CYP2E1*	Cytochrome P450, family 2, subfamily E, polypeptide 1	6.22		3.79
*ATP5B*	ATP synthase, H+ transporting, mitochondrial F1 complex	0.21		0.41
***FASN***	Sus scrofa fatty acid synthase	2.42		
*APOC3*	Sus scrofa apolipoprotein C-III		4.99	0.29
*CPT1A*	Sus scrofa carnitine palmitoyltransferase 1A		0.45	
***LPINI***	Sus scrofa lipin 1 mRNA	0.35	0.38	0.72
***PPARGC-1***	Sus scrofa peroxisome proliferator activated receptor gamma, coactivator 1 alpha (PPARGC-1)	2.37		
Glucose metabolic process				
*UGP2*	UDP-glucose pyrophosphorylase 2	0.16		0.40
*GPI*	Glucose-6-phosphate isomerase	0.20		0.34
*GPD1*	Glycerol-3-phosphate dehydrogenase 1	0.38		0.44
***PFKM***	Phosphofructokinase, muscle	0.36		0.45
*TPI1*	Triosephosphate isomerase 1	0.15		0.23
*PKM2*	Pyruvate kinase, muscle	0.14		0.23
*PGM1*	Phosphoglucomutase	0.35		0.33
***PCK1***	Sus scrofa phosphoenolpyruvate carboxykinase 1	2.42	2.42	
Muscle structure development				
*SHOC2*	Soc-2 suppressor of clear homolog	0.32		0.45
*CASQ1*	Calsequestrin 1 (fast-twitch, skeletal muscle)	0.46		0.46
*MYH4*	Myosin, heavy chain 4, skeletal muscle	0.29		0.43
*ACTA1*	Actin, alpha 1, skeletal muscle	0.32		0.36
*ACTA2*	Actin, alpha 2, smooth muscle,	0.39	0.40	
*ACTC1*	Actin, alpha, cardiac muscle 1	0.435	0.26	
*BIN1*	Bridging integrator 1	0.17		0.16
*HMGB1*	High mobility group box 1	0.45		0.34
*SGCA*	Sarcoglycan, alpha	0.25		0.40
*ITGB1*	Integrin, beta 1	0.18		0.27
*CTNNB1*	Catenin (cadherin-associated protein), beta 1	0.38		0.49
***MSTN***	Myostatin	0.41	1.14	0.36
***MYO1B***	Myosin IB	0.349	0.42	
*PPP3CB*	Protein phosphatase 3, catalytic subunit, beta isozyme	0.18		0.28
*CAV2*	Caveolin 2	0.25		0.45
*TPM2*	Tropomyosin 2 (beta)	0.33		0.36
***TNNT3***	Troponin T type 3 (skeletal, fast)	0.39		0.45
***TNNI1***	Troponin I type 1 (skeletal, slow)	0.37		
*LMNA*	Prelamin-A/C	0.27		0.44
*MYOT*	Sus scrofa myotilin	0.40	0.44	
*TPM3*	Sus scrofa tropomyosin 3	0.41		
*TNNI3*	Troponin I type 3	0.39	0.42	0.45
Cellular response to stress				
*GTF2H4*	General transcription factor IIH subunit 4	2.13		
*ERCC1*	Excision repair cross-complementing rodent repair complementation group 1	2.06		
***HSPA1L***	Heat shock 70 kDa protein 1-like	2.20		2.64
*MLH1*	mutL homolog 1	2.09		
*GADD45A*	Growth arrest and DNA-damage-inducible, alpha	2.04		
*CDKN1A*	Cyclin-dependent kinase inhibitor 1A			3.78
***MB***	Sus scrofa myoglobin (MB)	0.12		0.25
*CXCL10*	Chemokine (C-X-C motif) ligand 10			2.58
*MAPK14*	Mitogen-activated protein kinase 14	3.02		2.11
*TICAM2*	Toll-like receptor adaptor molecule 2	3.35		2.02
Cellular response to hormone stimulus				
*NR3C1*	Nuclear receptor subfamily 3, group C, member 1	0.48		
***GHR***	Growth hormone receptor	0.27		
*ACVR1*	Activin type I receptor	0.30		
*ADRB2*	Adrenergic, beta-2- receptor	0.32		0.49
*IFNGR2*	Interferon gamma receptor 2	0.27		
*FOLR2*	Folate receptor 2	0.47		0.44
*ADIPOR1*	Adiponectin receptor 1	0.48		

^a^Genes shown in boldface were used in qPCR verification

^b^Only the fold change of differentially expressed genes related to energy metabolism, muscle structure, and stress response are shown. Values > 1.0 show upregulation, and < 1.0 show downregulation

To further characterize the types of genes altered in response to heat stress or reduced feed intake, the differentially expressed genes were classified according to gene ontology (GO) slim terms. GO slim ranks high level terms from each of the three major gene ontologies: biological process, cellular component, and molecular function. Biological process analysis results are listed in Additional file 2: Tables S2, Additional file 3: Table S3, and Additional file 4: Table S4. Biological process analysis found that the differentially expressed genes were predominantly related to energy metabolism, muscle tissue development, catabolic process, or stress. Compared with the 22AL pigs, the downregulated genes in the 35AL pigs are mainly involved in muscle development or cell growth (GO:0060537, GO:0045927), actin cytoskeleton organization (GO:0030036), cellular ketone or oxoacid metabolic process (GO:0042180, GO:0043436), actin filament-based process or actomyosin structure organization (GO:0030029, GO:0031032), cellular component organization (GO:0016043), and catabolic process (GO:0009056); the upregulated genes are mainly involved in RNA processing or translation(GO:0006364,GO:0006412), DNA damage stimulus or DNA recombination (GO:0006974, GO:0016444), cell cycle arrest (GO:0022402), and amino acid metabolism (GO:0043038) (Additional file 2: Table S2). Assessing reduced feed intake, compared with 22AL controls, the downregulated genes in 22 PF pigs are mainly involved in muscle contraction or morphogenesis (GO:0006936, GO:0060415), cellular component biogenesis or organization (GO:0044085, GO:0016043,GO:0071840), and muscle cell differentiation (GO:0051146); the upregulated genes are mainly involved in response to nutrient levels (GO:0031667), response to extracellular stimulus (GO:0009991), lipid, steroid, or acetyl-CoA metabolic process (GO:0006629, GO:0008202, GO:0006084) (Additional file 3: Table S3). Assessing direct effects of heat stress, compared with 22PF pigs, the downregulated genes in the 35AL pigs are involved in muscle development (GO:0060537, GO:0061061), growth rate (GO:0040009, GO:0040010), glucose metabolism (GO:0006006, GO:0006096, GO:0006007), and catabolic process (GO:0009056, GO:0044282); the upregulated genes are involved in immune or heat stress response (GO:0006955, GO:0009408), negative regulation of cell cycle (GO:0045786), response to DNA damage stimulus or DNA recombination (GO:0006974, GO:0006310), negative regulation of catalytic activity (GO:0043086), and protein folding (GO:0006457) (Additional file 4: Table S4).

To define the biological pathways potentially associated with heat stress on meat quality or growth performance, KEGG pathway analysis (Table 3) revealed that, compared with the 22AL controls, the significantly upregulated pathways in 35AL were associated with ribosomes, p53 signaling pathway, and adipocytokine signaling pathway, whereas downregulated pathways were related to arginine and proline metabolism, glycolysis/gluconeogenesis, PPAR signaling, phagosome, and endocytosis pathways, citrate cycle (TCA cycle), dilated cardiomyopathy pathway, fatty acid metabolism, valine, leucine and isoleucine degradation, pyruvate metabolism, and leukocyte transendothelial migration pathway. Compared with the 22AL controls, metabolism of xenobiotics by cytochrome P450, bile secretion, and PPAR signaling pathway were upregulated in 22PF pigs and dilated cardiomyopathy, phagosome and antigen processing, and presentation were downregulated. The comparison between 35AL and 22PF groups of pigs identified direct effects of heat treatment, showing upregulation of adipocytokine signaling pathway, Herpes simplex infection, and Toll-like receptor signaling pathway, and downregulation of glycolysis/gluconeogenesis, citrate cycle (TCA cycle), hypertrophic cardiomyopathy (HCM), starch and sucrose metabolism, dilated cardiomyopathy, arginine, and proline metabolism pathways.

**Table 3:** Significant KEGG pathway analysis

Pathway name		Gene involved	*p*	FDR	Genes
22PF vs 22AL (up)					
Up	Metabolism of xenobiotics by cytochrome P450	2/53	0.0110	0.782	*CYP2E1*//*SULT2A1*
	Bile secretion	2/59	0.0136	0.782	*OCT1*//*SULT2A1*
	PPAR signaling pathway	2/64	0.0158	0.782	*APOC3*//*PCK1*
Down	Dilated cardiomyopathy	5/41	0.0003	0.0483	*ACTC1*//*MLC2V*//*PLN*//*TNNI3*//*TPM3*
	Phagosome	6/134	0.0003	0.0483	*GP91-PHOX*//*SLA-5*//*SLA-DQA1*//*SLA-DRB1*//*THBS1*//*THBS3*
	Antigen processing and presentation	4/63	0.0011	0.054	*CD8A*//*SLA-5*//*SLA-DQA1*//*SLA-DRB1*
35AL vs 22AL					
Up	Ribosome	6/83	0.0008	0.13	*RPL10A*//*RPL11*//*RPL14*//*RPLP1*//*RPS17*//*RPS4*
	p53 signaling pathway	5/60	0.0011	0.13	*BID*//*CDKN1A*//*FAS*//*GADD45A*//*IGF1*
	Adipocytokine signaling pathway	3/61	0.0468	1	*PCK1*//*POMC*//*PPARGC-1*
Down	Arginine and proline metabolism	11/47	3.09E-07	7.64E-05	*ACY1*//*ALDH2*//*ARG2*//*CKM*//*CKMT2*//*GATM*//*GLUD1*//*GLUL//GOT1*//*GOT2*//*P4HA1*
	Glycolysis/gluconeogenesis	9/51	4.38E-05	0.0024	*ACSS2*//*ALDH2*//*DLAT*//*DLD*//*GPI*//*PFKM*//*PGM1*//*PKM2*//*TPI1*
	PPAR signaling pathway	10/64	4.92E-05	0.0024	*ACADL*//*ACADM*//*ACOX1*//*ACSL1*//*CD36*//*CPT1B*//*CYP27A1*//*FABP3*//*LPL*//*SCD*
	Phagosome	14/134	0.0002	0.0056	*ACTB*//*ACTB*//*ATP6V1D*//*ATP6V1G1*//*CD36*//*EEA1*//*ITGAV*//*ITGB1*//*M6PR*//*SLA-1*//*SLA-3*//*SLA-5*//*SLA-DQA1*//*SLA-DRB1*
	Endocytosis	16/179	0.0003	0.0081	*ADRB2*//*CAV1*//*CAV2*//*CXCR4*//*EEA1*//*EGF*//*EPS15*//*FOLR2*//*MDM2*//*MET*//*SLA-1*//*SLA-3*//*SLA-5*//*TGFB2*//*USP8*//*WWP1*
	Citrate cycle (TCA cycle)	6/29	0.0004	0.0081	*DLAT*//*DLD*//*FH*//*IDH2*//*IDH3A*//*SDHA*
	Dilated cardiomyopathy	10/81	0.0004	0.0081	*ACTB*//*ACTB*//*ACTC1*//*DES*//*ITGAV*//*ITGB1*//*LMNA*//*SGCA*//*TGFB2*//*TPM2*
	Fatty acid metabolism	7/41	0.0004	0.0081	*ACADL*//*ACADM*//*ACOX1*//*ACSL1*//*ALDH2*//*CPT1B*//*LCTHIO*
	Valine, leucine and isoleucine degradation	7/44	0.0006	0.0117	*ACADM*//*ALDH2*//*BCKDHA*//*BCKDHB*//*DLD*//*LCTHIO*//*PCCB*
	Pyruvate metabolism	6/36	0.0012	0.0194	*ACSS2*//*ALDH2*//*DLAT*//*DLD*//*GLO1*//*PKM2*
	Leukocyte transendothelial migration	10/103	0.0024	0.0354	*ACTB*//*ACTB*//*CDH5*//*CTNNB1*//*CXCL12*//*CXCR4*//*F11R*//*ITGB1*//*MAPK14*//*RAP1A*
35AL vs 22PF (up)					
Up	Adipocytokine signaling pathway	3/60	1.6E-06	0.0004	*PCK1*//*POMC*//*PPARGC-1*
	Herpes simplex infection	8/157	0.0002	0.0193	*CASP3*//*CCL5*//*OAS1*//*OAS2*//*PTPN11*//*SLA*//*SOCS3*//*STAT2*
	Toll-like receptor signaling pathway	6/87	0.0002	0.0193	*CCL5*//*CD40*//*CXCL10*//*MAPK14*//*TICAM2*//*TLR1*
Down	Glycolysis/gluconeogenesis	7/51	1.97E-05	0.0049	*DLD*//*ENO3*//*GPI*//*PFKM*//*PGM1*//*PKM2*//*TPI1*
	Citrate cycle (TCA cycle)	5/29	0.0001	0.0131	*DLD*//*FH*//*IDH2*//*IDH3A*//*MDH2*
	Hypertrophic cardiomyopathy (HCM)	7/76	0.0003	0.0166	*CACNA2D1*//*DES*//*ITGB1*//*LMNA*//*SGCA*//*TGFB3*//*TPM2*
	Starch and sucrose metabolism	5/35	0.0003	0.0166	*AMY2*//*GPI*//*PGM1*//*PYGM*//*UGP2*
	Dilated cardiomyopathy	7/81	0.0004	0.0194	*CACNA2D1*//*DES*//*ITGB1*//*LMNA*//*SGCA*//*TGFB3*//*TPM2*
	Arginine and proline metabolism	5/47	0.0011	0.0381	*ACY1*//*CKM*//*GLUD1*//*GOT1*//*MAOB*
	PPAR signaling pathway	4/64	0.022	1.6496	*ACADM*//*ACSL1*//*APOC3*//*CYP27A1*

### Verification of mRNA array analyses with qPCR

**Fig. 2:**
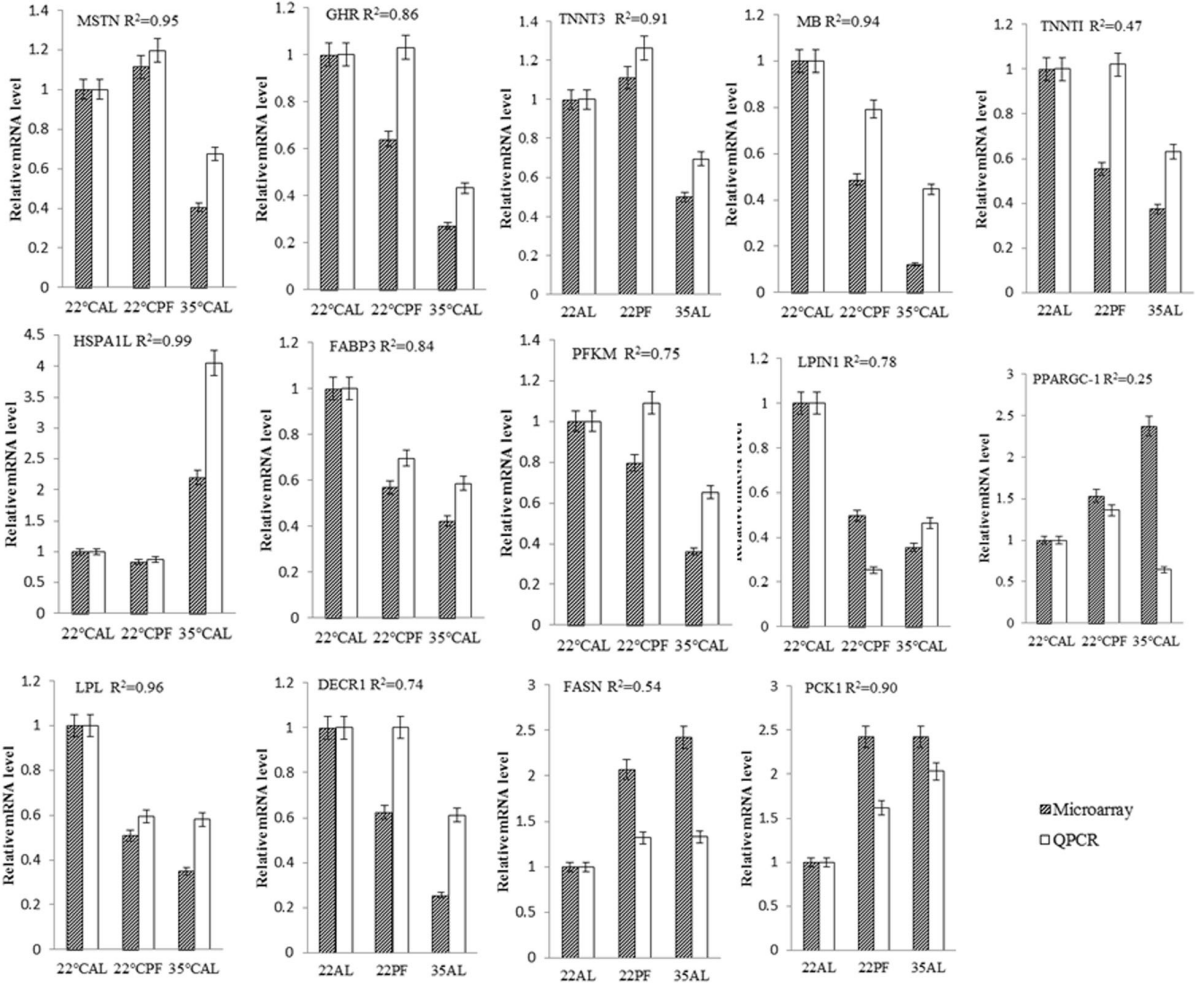
Correlations between relative abundance of selected transcripts determined by qPCR and gene chips. Vertical axis, relative abundance, normalized to values in pigs fed ad libitum at 22 °C. Correlations are between gene chips and qPCR pairs of data (*n* = 4 pools of two pigs per treatment)

A selection of 14 genes with relatively large change and related to muscular energy metabolism or muscle development, *DECR1*, *FABP3*, *GHR*, *HSPA1L*, *LPIN1*, *LPL*, *MB*, *MSTN*, *PFKM*, *PPARGC1*, *TNNI1*, *TNNT3*, *FASN*, and *PCK1* (see Table 2), were chosen to verify that their relative transcript abundance determined by the gene-chips could be confirmed by real-time PCR (qPCR). With the exception of *PPARGC1*, there were high correlations between the two methods (Fig. 2).

**Fig. 3:**
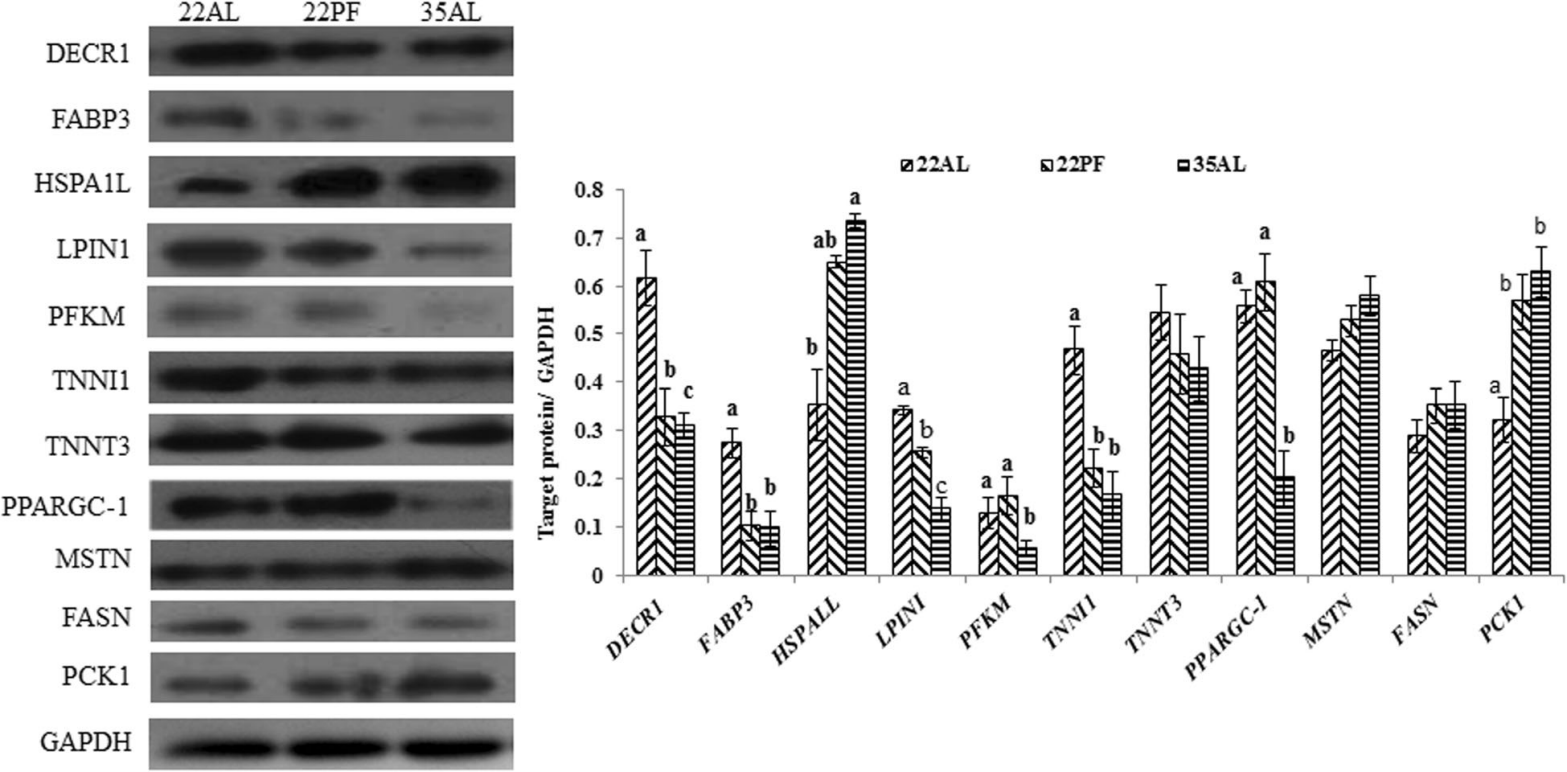
Western blots for a selection of differentially expressed proteins. Targeted protein DECR1, FABP3, HSPA1L, LPIN1, PFKM, TNNT3, TNNI1, PPARGC-1, MSTN, FASN, and PCK1, with GAPDH as a reference protein. **a** Representative sample from each treatment fractionated by SDS-PAGE, blotted and then detected with specific antibodies. **b** Semi-quantification of protein content from scans of band, (*n* = 4 pools of two pigs per treatment)

Based on these results, 11 proteins (DECR1, FABP3, HSPAL1, LPIN1, PFKM, TNNT3, TNNI1, PPARGC-1, MSTN, FASN, and PCK1) were selected and correspondence between protein abundance, by Western blots, and transcript abundance, by qPCR, was examined. The results (Fig. 3) showed that the relative transcript abundance of 13 genes (*DECR1*, *FABP3*, *GHR*, *HSPA1L*, *LPIN1*, *LPL*, *MB*, *MSTN*, *PFKM*, *TNNT1*, *TNNT3*, *FASN*, and *PCK1*) had high correlations to the mRNA array result. While *PPARGC1* had lower correlations to the mRNA array result. Among 11 proteins, the DECR, FABP3, LPINI, and TNNT1protein levels were downregulated and PCK1 protein level was upregulated in 35AL and 22PF group compared with the control, which were correspondent with their mRNA result, but TNNT3, MSTN, and FASN protein levels in 35AL group and 22PF group were not different from that in the 22AL group, which were not correspondent with their mRNA result. HSPAL1 protein level was upregulated and PFKM was downregulated in 35AL compared with 22PF and 22AL group, which were correspondent with their mRNA result. Both PPARGC-1 protein level and mRNA level in 35AL group were downregulated, which is not consistent with the mRNA array result (Table 4).

**Fig. 4:**
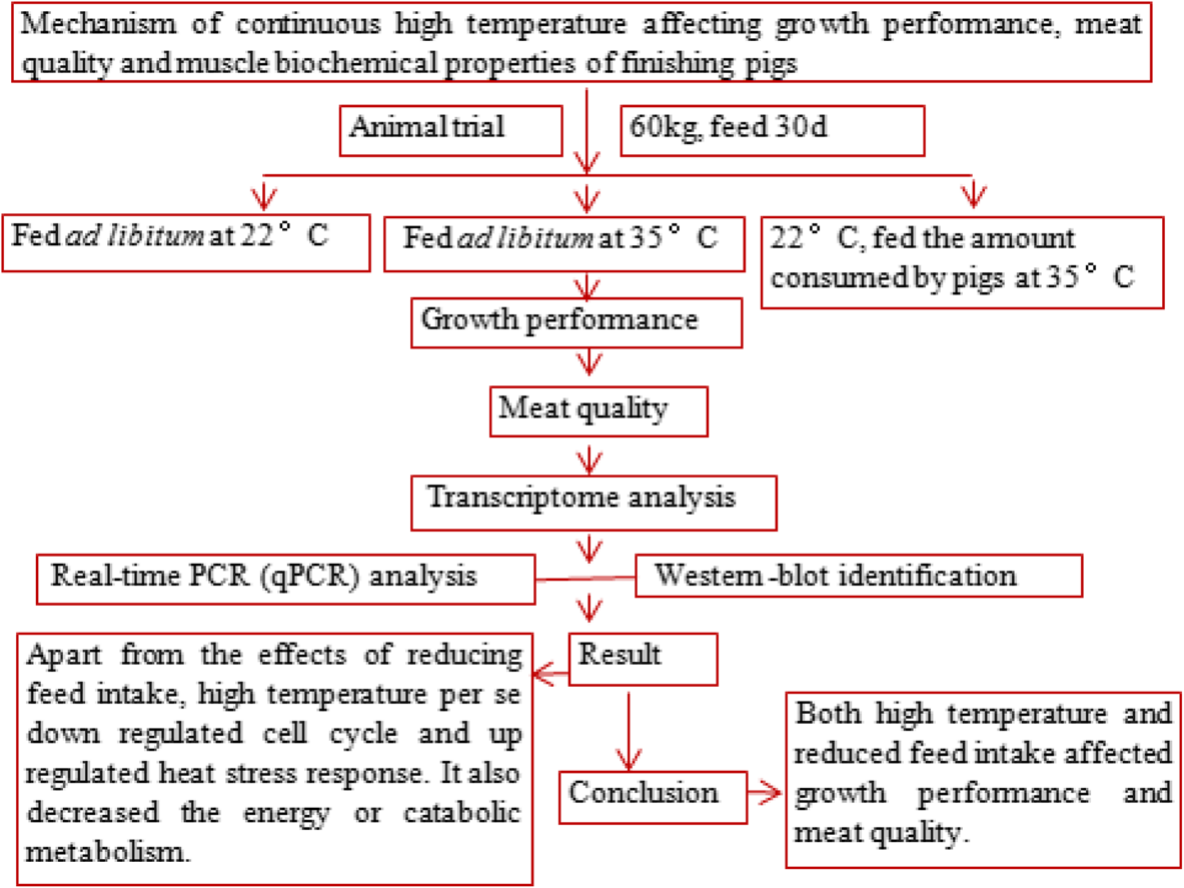
TOC graphic of this article

**Table 4:** Primer sequences

Target gene	Primer sequence (5′ to 3′)	Accession no.	Product size (bp)
*FABP3*	Forward: CGCCTGTTCTGTCGTCTC	NM_001099931.1	263
	Reverse: TCTCATCAAACTCCACTCCC		
*GHR*	Forward: TGCCCAGGTAAGCGACAT	NM_214254.2	272
	Reverse: TGCCAGCAGTAGTGGTAAGG		
*HSPA1L*	Forward: CCATGAATCCCCAGAACACT	NM_001123128.1	313
	Reverse: ATGACGCCTGCATCCTTGGT		
*Lpin1*	Forward: TTCTCACAACACTGACCCTCTT	EU164847	330
	Reverse: GCCCTCCGACTCTAACCAC		
*MB*	Forward: CCCGAGACCCTGGAGAAAT	NM_214236.1	457
	Reverse: CAACACTCAGAAGCAAACCCTAC		
*MSTN*	Forward: AATGAGAACAGCGAGCAA	AY448008.2	279
	Reverse: TTCCGTCGTAGCGTGATA		
*PFKM*	Forward: ACCGAGTGCTGGTTGTGC	NM_001044550.1	305
	Reverse: GTTGTTGGAGACCGTGGC		
*PPARGC-1*	Forward: AAAGCCAACCAAGATAACCC	NM_213963.1	242
	Reverse: TCACCAAATAGCCGCAGAC		
*LPL*	Forward: CGACTGGATGGCGGTTG	NM_214286	271
	Reverse: ATGCCCTACTGGTTTCTGGAT		
*TNNT3*	Forward: CCCTCATCGACAGCCACTT	NM_001001863.1	318
	Reverse: GCACCTTCTTCTTCATCTCCC		
*TNNI1*	Forward: TGAACGACCTCCAGAAAGC	NM_213912.3	345
	Reverse: GGCGGACACTCAGGAATAA		
*DECR1*	Forward: TAAAGCACAGAAAGGAGCAG	NM_001190232.2	217
	Reverse: TCCAGTTGGGTCAAGACG		
*FASN*	Forward: CCTCATCGGCGGTGTGGA	NM_004104	122
	Reverse: TGAAGTCGAAGAAGAAGGAGAGC		
*PCK1*	Forward: ACAGCCTGCCCCAGGC	NM_001123158	148
	Reverse: TTCAGCCTCTTGATGAC		
*18S RNA*	Forward: GAGCGAAAGCATTTGCCAAG	AF102857	101
	Reverse: GGCATCGTTTATGGTCGGAAC		

## Discussion

The present study showed that high temperature decreased the growth performance, which is consistent with others’ findings [1–3]. Le Dividich et al. [6] reported that high temperature decreased feed intake and that decreased energy intake was the main reason for the decreased IMF deposition. The restricted feed intake from exposure here to high temperature also decreased IMF content, indicating that the effect of high temperature on IMF content was explained mainly by the decreased feed intake. In addition, many genes related with lipid metabolism such as FABP3, LPL, SCD, DECR, and LPINI were downregulated, which decreased the fat synthesis and deposition. High temperature also increased pH value and decreased the content of glycogen in muscle, which is consistent with Hu et al. [15]. The shear force in feed-restricted group is greater compared to the control group, because of the lower intramuscular fat. As high temperature decreased the calpastatin expression [16] and possibly enhancing post-mortem proteolysis to offset any decreased tenderness from the lower IMF content, the shear force in higher temperature is not different from the control.

In this study, mRNA array analysis of the LM was used to explore the mechanisms of underlying the effects of high temperature on meat quality and growth performance. High temperature has previously been shown to cause DNA damage [16, 17] and protein mis-folding [18], heat stress increased the expression of heat shock proteins, especially the heat shock protein 70 family [19, 20]. In the present study, the GO analysis showed that high temperature per se upregulated gene expression involved in stress reaction in cells, including *GTF2H4*, *ERCC1*, *HSPA1L*, *POLL*, mutL homolog 1 (*MLH1*), *GADD45A*, and *CDKN1A*. *HSPAL1* belong to 70-kDa heat shock proteins (Hsp70s), which have housekeeping functions and assist a wide range of folding processes, including the folding and assembly of newly synthesized proteins, refolding of mis-folded and aggregated proteins, membrane translocation of cellular and secretory proteins, and control of the activity of regulatory proteins [21, 22]. In addition, heat shock proteins also adjust redox balance and relieve oxidative stress through inhibiting the activity of NADPH oxidase [23], which was one of the important reasons for high temperature causing oxidative stress. Compared with the 22PF pigs, the 35AL animals had increased expression of heat shock protein 90 (*HSPCB*), heat shock protein 90 (*HSPH1*). The *GTF2H4* and *ERCC1* genes also are involved in DNA repair-related biological processes, reflecting an enhanced self-protecting mechanism under high temperature stress. In addition, genes of ncRNA and rRNA metabolism, protein translation, and translation extension also were upregulated, which might be required by synthesis of heat stress proteins.

Heat stress also affected expression of some genes related to muscle structure. Many studies have reported that high temperature decreased carcass muscle weight [2, 24, 25]. High temperature here decreased the relative expression of actin protein genes in LM including *ACTA2*, *ACTC1*, and *ACTA1*, myosin heavy chain IIb (*MYH4*), myofibrillar protein connection (*DES*), the connection protein with the membrane and myofibrils (*SGCA*), troponins*TNNT3* and*TNNI1*, nest bridge proteins (*CAV2*, *3*), β-Tropomyosin (TPM2), and Integrinβ1(*ITGB1*), all involved in muscle structure, fiber development, or muscle contraction. In addition, the high-temperature treatment induced a fiber transformation from slow type to fast type and downregulated CaMK and PPAR signaling pathways, but limited feed intake did not. The change in fiber type is largely caused by reduced CaMK and PPAR cell signaling. For example, Gibala et al. [26] reported that over-expression of PPAR-γ induced a cell transformation from type II to type I. High temperature decreased muscle development here, independently of the reduced feed intake.

High temperature also affected expression of the genes related to energy metabolism. Rinaldo and Le Dividich [7] previously reported that high temperature decreased key enzymes related to oxidative and glycolytic metabolisms including lactate dehydrogenase, beta-hydroxy coenzyme A dehydrogenase, citrate synthase, and cytochrome oxidase. Weller et al [10] then reported that high temperature (34 °C) decreased gene expression of NADH dehydrogenase 1 (*ND1*), NADH dehydrogenase 2 (*ND2*), cytochrome C oxidase complex, *ATP5*, *ATP6*, and others involved in the electron transport chain. These results implied that long-term exposure to high temperature reduced the level of energy metabolism in muscle. Consistent with those findings, high temperature here decreased expression of phosphofructokinase (*PFKM*), glucose-6-phosphate isomerase (*GPI*), pyruvate kinase (*PKM2*), triosephosphateisomerase 1 (*TPI1*), isocitrate dehydrogenase 3 (*IDH3A*), and others involved in carbohydrate metabolism, glucose metabolism, and other biological processes. Downregulated expression of these genes indicated that the g glycolytic and oxidative metabolisms were decreased under high temperature environment, and this would be expected to affect meat quality.

High temperature also downregulated genes involved in the TCA cycle, likely indicating reduced total energy metabolism in muscle. It also reduced expression of genes involved in ATP and amino acid synthesis and decomposition. These changes likely provide an adaptive, reduced thermogenic response to heat stress. In the amino acid synthesis and decomposition category, aspartate amino-transferase (GOT) and creatine kinase (CKM) both relate to intramuscular energy, creatine, and ADP phosphoexchange, and therefore generation of hydrogen ions determining postmortem pH [27]. Kwasiborski et al. [28] reported that high levels of CKM in muscle lead to an increase in the rate of muscle metabolism within the early stages after slaughter, so that ultimate pH is lower. High temperature here decreased the *CKM* expression, which may be a reason for the higher pH. Related to decrease the content of IMF, high temperature altered expression of genes involved in lipid metabolism. For example, catalytic subunit A (*PPP3CB*), signal transducer and activator of transcription 5A (*STAT5A*), and *ATP5B* synthase beta subunit (*ATP*) were downregulated in animals exposed to high temperature, as were fatty acid binding protein 3 (*FABP3*), long-chain acyl coenzyme A dehydrogenase (*ACADL*), medium-chain acyl coenzyme A dehydrogenase (*ACADM*), palmitoyl coenzyme A oxidase (*ACOX1*), Ketoacyl CoA Thiolase (*LCTHIO*), and carnitine acyl transfer enzyme 1B (*CPT1B*), involved in fatty acid location and beta oxidation-related processes. A decline in oxidative capacity may be another adaptation to high temperature by reducing thermogenesis. Wu et al. [29] found that high temperature decreased IMF content by decreasing the activities of acetyl coenzyme A (ACC) and fatty acid synthase (FAS); it also inhibited beta-oxidation of fatty acids by decreasing the hydroxyacyl CoA dehydrogenase (HAD) in skeletal muscle. FASN, involved in fatty acid synthesis, was upregulated in mRNA array data, but qPCR and Western-blot in this present research showed that there were no differences on FASN protein among three groups, probably the fatty acid synthesis try to compensate for too lower IMF.

In the 22PF animals, the effects of reduced intake were separated from heat-stress per se; genes involved in fatty acid decomposition including *DECR1*, *LPIN1*, FABP3, and *CPT1A* were downregulated; the GO analysis result showed that muscle cell development and cellular component biogenesis also were downregulated. Some genes related to lipid metabolite such apolipoprotein C3 (*APOC3*), cytochrome P4502C34 (*CYP2C34*), P450 2E1 (*CYP2E1*), sulfate transferase 2A1 (*SULT2A1*), phosphatidylserine decarboxylase proenzyme (PISD), serum retinol binding protein 4 (*RBP4*), and PEP carboxykinase1 (*PCK1*) were not influenced by restricted feed intake. Although response to nutrient levels, vitamins and proteins, and lipid metabolic process were upregulated, the growth performance and IMF were lower than in 22AL group, which implies that the nutrient or energy intake did not meet the need of the pigs, and the pigs try to synthesize fat or other nutrients, but the nutrient and energy were too low to satisfy the needs of the pigs. While *APOC3*, *CYP2E1*, *SULT2A*, and *PISD* were downregulated in animals exposed to high temperature and GO analysis showed that heat stress, DNA damage, and negative regulation of cell cycle were upregulated, this implied that high temperature damaged the cell function and cannot synthesize the nutrients; pigs in 35AL group did not response to the nutrient level, but did response to the heat stress. This finding clearly shows differences between high temperature and limited feed intake in their effects on transcripts related to energy metabolism in muscle.

The most imported signaling pathways, identified by KEGG, are discussed below.

### p53 signaling pathway

Skeletal muscle atrophy is reflected in the number of nuclei [30], reduced mainly through apoptosis and the p53 signaling pathway, activated by stress signals including DNA damage, oxidative stress, and induction and activation of cancer genes. The p53 protein regulates transcriptional activation of many genes, mainly involved in cell cycle arrest, cell senescence, and apoptosis [31]. Nitta et al. [32] reported that high temperature arrested cell cycles depending upon the p53 signaling pathway. Exposure of pigs to high temperature in the present study increased expression of genes of the p53 signaling pathway, apoptosis, and skeletal muscle atrophy, but p53 signal pathway was just upregulated in 35ALgroup compared with 22AL and 22PFgroup, which implied that the upregulation of p53 signal pathway was a direct temperature effect. Genes such as *p21* (*CDKN1A*), *Bid* and *FasFADD*, *Caspase 8*, and *Caspase10* were involved and are known to induce apoptosis [33]. Signaling complexes, including *FADD*, *Caspase 8*, and*Caspase10*, also induced apoptosis [33]. Yamada et al. [34] reported that human fast muscle type may be more likely to be induced to apoptosis and the same is true for rat [35]. So it can be deduced that high temperature induced more fast muscle type production through up-regulating the p53 pathway.

### PPAR signaling pathway

PPAR-alpha and PPAR-sigma, members of the nuclear hormone receptor super family [36], influence gene transcription of fatty acid oxidation enzymes, such as*FABP3*, *CPT1*, and *ABCA1* in skeletal muscle [37–39]. In this present study, genes of the PPAR signal pathway were downregulated in pigs exposed to high temperature, as were *ACADL*, *ACADM*, *ACOX1*, *ACSL1*, and *CPT1B*, all related to fatty acid beta-oxidation. It also is downregulated in 35AL compared with 22PF, but the FDR is very high (FDR = 1.64), the FDR of PPAR signaling pathway in 22PF vs 22AL also is very high (FDR = 0.78). It was deduced, therefore, that high temperature decreased energy metabolism through the PPAR signaling pathway.

### Adiponectin signal pathway

Adiponectin is an adipocytokine playing an important role in insulin sensitivity and glucose and lipid metabolism, especially in skeletal muscle; it activates AMPK, p38MAPK, and PPAR signal pathway [40] and affects fatty acid metabolism. Adipocytokine signaling pathway also was involved in adaptive responses to heat stress [19], which is important regulators of energy homeostasis, food intake, and insulin action. In the present research, adiponectin signaling pathway was downregulated in muscle of pigs exposed to high temperature, which improved energy expenditure as adaptive response to heat stress.

Combining the results of GO and KEGG pathway analyses, the genes downregulated by reduced feed intake were mainly involved in muscle contraction, muscle development, muscle system process, or differentiation, while the upregulated genes were mainly involved in response to nutrient levels or extracellular stimuli. Downregulated genes caused by high temperature were mainly involved in muscle structure and development, energy, or catabolic metabolism, while upregulated genes were mainly involved in DNA or protein damage or recombination, or processes of cell cycle, biogenesis, and stress and immune responses. The comprehensive analyses of the transcriptome of porcine skeletal muscle provided here indicate some of the molecular basis for direct effects of exposure to high temperature on traits related to meat quality, distinct from indirect effects resulting from depressed feed intake.

## Conclusions

Both high temperature and reduced feed intake affected growth performance and meat quality. Apart from the effects of reducing feed intake, a direct effect of high temperature on growth performance and meat quality also was involved in negatively regulating cell cycle, stimulating protein, DNA damage and cell apoptosis, and heat stress response. High temperature also decreased the energy or catabolic metabolism level through PPAR signaling pathway (Fig. 4).

## Additional files

Additional file 1: **Table S1.** Ingredient 7 composition and nutrient content of the basal diets (DOCX 14 kb)

Additional file 2: **Table S2.** Go analysis of differently expressed genes in35AL group compared with 22AL group (DOCX 15 kb)

Additional file 3: **Table S3.** GO analysis of differently expressed genes in 22PF group compared with 22AL group (DOCX 15 kb)

Additional file 4: **Table S4.** GO analysis of differently expressed genes in 35AL group compared with 22PF group (DOCX 15 kb)

## Abbreviations

ADFI: Average daily feed intake
ADG: Average daily gain
AL: Ad libitum
BW: Body weight
F:G: Feed to gain
HCM: Hypertrophic cardiomyopathy
IMF: Intramuscular fat
LM: Longissimus muscle
PF: Pair fed
PPAR: Peroxisome proliferator activated receptor
RT: Room temperature
