# Supplementary material for: Mechanism of continuous high temperature affecting growth performance, meat quality, and muscle biochemical properties of finishing pigs
Source: Genes Nutr. 2019 Jul 24;14:23. doi: 10.1186/s12263-019-0643-9 (PMC6657146; doi:10.1186/s12263-019-0643-9)
Supplement: Supplementary file 1 — Table S1. Ingredient 7 composition and nutrient content of the basal diets (DOCX 14 kb) [file 12263_2019_643_MOESM1_ESM.docx]

Supplemental table 1 Ingredient composition and nutrient content of the basal diets

| Ingredients | % | Nutrient level |  |
| --- | --- | --- | --- |
| Corn meal | 77.37 | ME(Kcal/kg) | 3314.55 |
| Soybean meal | 19.56 | CP(%) | 15.71 |
| *Premix | 1 | Calcium(%) | 0.59 |
| Lime powder | 0.92 | Total P(%) | 0.49 |
| Dicalcium phosphate | 0.75 | Available P(%) | 0.2 |
| Salt | 0.3 | Met(%) | 0.24 |
| Lysine | 0.1 | Lys(%) | 0.77 |
|  |  | Met+Cys(%) | 0.49 |
|  |  | Thr(%) | 0.51 |
|  |  | Trp(%) | 0.15 |
|  |  | Val(%) | 0.65 |

*Providing 1,750 IU/kg vitamin A, 220 IU/kg vitamin D3, 3 IU/kg vitamin E, 0.55 mg/kg vitamin K3, 0.25 mg/kg vitamin B1, 1.0 mg/kg vitamin B2, 0.7 mg/kg vitamin B6, 3 lg/kg vitamin B12, 4 mg/kg niacin, 1.6 mg/kg calcium pantothenate, 0.1 mg/kg folic acid, 7lg/kg biotic, 0.08 g/kg choline chloride, 6.5 mg/kg manganese, 15 mg/kg iron, 15 mg/kg zinc, 1.5 mg/kg copper, 0.07 mg/kg iodine(I2), 0.03 mg/kg selenium, and 1 g/kg sodium chloride
