# Supplementary material for: Mechanism of continuous high temperature affecting growth performance, meat quality, and muscle biochemical properties of finishing pigs
Source: Genes Nutr. 2019 Jul 24;14:23. doi: 10.1186/s12263-019-0643-9 (PMC6657146; doi:10.1186/s12263-019-0643-9)
Supplement: Supplementary file 2 — Table S2. Go analysis of differently expressed genes in35AL group compared with 22AL group (DOCX 15 kb) [file 12263_2019_643_MOESM2_ESM.docx]

Supplemental table 2. Go analysis of differently expressed genes in35AL group compared with 22AL group

| GO ID | GO item | Counts | *P* value |
| --- | --- | --- | --- |
| Up-regulated (top15) | | | |
| GO:0006412 | translation | 13 | 5.90E-05 |
| GO:0034660 | ncRNA metabolic process | 7 | 2.28E-04 |
| GO:0006364 | rRNA processing | 5 | 2.53E-04 |
| GO:0042254 | ribosome biogenesis | 6 | 2.56E-04 |
| GO:0071843 | cellular component biogenesis at cellular level | 7 | 5.13E-04 |
| GO:0006396 | RNA processing | 11 | 5.57E-04 |
| GO:0008152 | metabolic process | 84 | 2.00E-03 |
| GO:0022613 | ribonucleoprotein complex biogenesis | 6 | 2.02E-03 |
| GO:0006418 | tRNA aminoacylation for protein translation | 3 | 3.56E-03 |
| GO:0043038 | amino acid metabolism | 3 | 3.56E-03 |
| GO:0002285 | lymphocyte activation involved in immune response | 4 | 5.31E-03 |
| GO:0002200 | somatic diversification of immune receptors | 3 | 7.88E-03 |
| GO:0016444 | somatic cell DNA recombination | 3 | 7.88E-03 |
| GO:0022402 | cell cycle arrest | 14 | 9.76E-03 |
| GO:0006974 | response to DNA damage stimulus | 8 | 1.25E-02 |
| Down-regulated (top 15) | | | |
| GO:0060537 | muscle tissue development | 35 | 1.39E-09 |
| GO:0030036 | actin cytoskeleton organization | 34 | 6.25E-09 |
| GO:0042180 | cellular ketone metabolic process | 53 | 1.20E-08 |
| GO:0019752 | carboxylic acid metabolic process | 52 | 1.40E-08 |
| GO:0043436 | oxoacid metabolic process | 52 | 1.40E-08 |
| GO:0030029 | actin filament-based process | 34 | 2.63E-08 |
| GO:0006082 | organic acid metabolic process | 52 | 3.28E-08 |
| GO:0045927 | positive regulation of growth | 32 | 4.10E-08 |
| GO:0031032 | actomyosin structure organization | 16 | 2.35E-07 |
| GO:0046907 | intracellular transport | 43 | 9.07E-07 |
| GO:0030239 | myofibril assembly | 14 | 1.36E-06 |
| GO:0007010 | cytoskeleton organization | 41 | 1.76E-06 |
| GO:0016043 | cellular component organization | 122 | 3.32E-06 |
| GO:0009056 | catabolic process | 60 | 3.48E-06 |
| GO:0044281 | small molecule metabolic process | 85 | 3.75E-06 |

Note: GO analysis is based on the Biological Process categories.
