# Supplementary material for: Mechanism of continuous high temperature affecting growth performance, meat quality, and muscle biochemical properties of finishing pigs
Source: Genes Nutr. 2019 Jul 24;14:23. doi: 10.1186/s12263-019-0643-9 (PMC6657146; doi:10.1186/s12263-019-0643-9)
Supplement: Supplementary file 3 — Table S3. GO analysis of differently expressed genes in 22PF group compared with 22AL group (DOCX 15 kb) [file 12263_2019_643_MOESM3_ESM.docx]

Supplemental table 3. GO analysis of differently expressed genes in 22PF group compared with 22AL group

| GO ID | GO item | Counts | *P* value |
| --- | --- | --- | --- |
| Up-regulated (top 15) | | | |
| GO:0031667 | response to nutrient levels | 9 | 1.13E-04 |
| GO:0009991 | response to extracellular stimulus | 9 | 1.75E-04 |
| GO:0009725 | response to hormone stimulus | 10 | 1.82E-03 |
| GO:0006629 | lipid metabolic process | 9 | 1.84E-03 |
| GO:0043434 | response to peptide hormone stimulus | 6 | 4.49E-03 |
| GO:0002637 | regulation of immunoglobulin production | 2 | 4.83E-03 |
| GO:0009719 | response to endogenous stimulus | 10 | 5.11E-03 |
| GO:0033273 | response to vitamin | 4 | 1.12E-02 |
| GO:0008202 | steroid metabolic process | 4 | 1.30E-02 |
| GO:0050714 | positive regulation of protein secretion | 2 | 1.45E-02 |
| GO:0002377 | immunoglobulin production | 2 | 1.55E-02 |
| GO:0006084 | acetyl-CoA metabolic process | 2 | 1.55E-02 |
| GO:0006094 | gluconeogenesis | 2 | 1.65E-02 |
| GO:0006775 | fat-soluble vitamin metabolic process | 2 | 1.65E-02 |
| GO:0019319 | hexose biosynthetic process | 2 | 1.65E-02 |
| Down-regulated (top 15) | | | |
| GO:0060415 | muscle tissue morphogenesis | 5 | 5.20E-06 |
| GO:0006936 | muscle contraction | 10 | 1.46E-05 |
| GO:0003012 | muscle system process | 10 | 3.32E-05 |
| GO:0055007 | muscle cell differentiation | 5 | 9.09E-05 |
| GO:0048646 | anatomical structure formation involved in morphogenesis | 17 | 9.67E-05 |
| GO:0055001 | muscle cell development | 8 | 1.01E-04 |
| GO:0044085 | cellular component biogenesis | 19 | 1.23E-04 |
| GO:0071842 | cellular component organization at cellular level | 27 | 1.62E-04 |
| GO:0009653 | anatomical structure morphogenesis | 29 | 2.47E-04 |
| GO:0016043 | cellular component organization | 33 | 2.69E-04 |
| GO:0055002 | striated muscle cell development | 7 | 3.96E-04 |
| GO:0071840 | cellular component organization or biogenesis | 33 | 4.22E-04 |
| GO:0051146 | striated muscle cell differentiation | 8 | 5.28E-04 |
| GO:0030239 | myofibril assembly | 5 | 6.12E-04 |
| GO:0048513 | organ development | 31 | 6.34E-04 |

Note: GO analysis is based on the Biological Process categories.
