# Supplementary material for: Mechanism of continuous high temperature affecting growth performance, meat quality, and muscle biochemical properties of finishing pigs
Source: Genes Nutr. 2019 Jul 24;14:23. doi: 10.1186/s12263-019-0643-9 (PMC6657146; doi:10.1186/s12263-019-0643-9)
Supplement: Supplementary file 4 — Table S4. GO analysis of differently expressed genes in 35AL group compared with 22PF group (DOCX 15 kb) [file 12263_2019_643_MOESM4_ESM.docx]

Supplemental table 4. GO analysis of differently expressed genes in 35AL group compared with 22PF group

| GO ID | GO item | Counts | *P* value |
| --- | --- | --- | --- |
| Up-regulated (top 15) | | | |
| GO:0006955 | immune response | 16 | 2.76E-04 |
| GO:0009408 | response to heat | 6 | 1.02E-03 |
| GO:0007050 | cell cycle arrest | 5 | 1.48E-03 |
| GO:0002376 | immune system process | 22 | 2.65E-03 |
| GO:0045786 | negative regulation of cell cycle | 6 | 2.95E-03 |
| GO:0006310 | DNA recombination | 4 | 3.54E-03 |
| GO:0030216 | keratinocyte differentiation | 3 | 4.96E-03 |
| GO:0002687 | positive regulation of leukocyte migration | 3 | 6.53E-03 |
| GO:0006974 | response to DNA damage stimulus | 7 | 6.68E-03 |
| GO:0002684 | positive regulation of immune system process | 9 | 8.79E-03 |
| GO:0006457 | protein folding | 4 | 1.07E-02 |
| GO:0009266 | response to temperature stimulus | 6 | 1.24E-02 |
| GO:0007049 | cell cycle | 13 | 1.62E-02 |
| GO:0030888 | regulation of B cell proliferation | 2 | 1.84E-02 |
| GO:0043086 | negative regulation of catalytic activity | 7 | 1.89E-02 |
| Down-regulated (top 15) | | | |
| GO:0007275 | multicellular organismal development | 97 | 1.71E-07 |
| GO:0044275 | cellular carbohydrate catabolic process | 10 | 1.22E-06 |
| GO:0040009 | regulation of growth rate | 14 | 7.55E-06 |
| GO:0060537 | muscle tissue development | 19 | 8.58E-06 |
| GO:0009056 | catabolic process | 36 | 2.46E-05 |
| GO:0016052 | carbohydrate catabolic process | 10 | 2.48E-05 |
| GO:0061061 | muscle structure development | 23 | 2.54E-05 |
| GO:0040010 | positive regulation of growth rate | 13 | 2.94E-05 |
| GO:0006091 | generation of precursor metabolites and energy | 14 | 4.97E-05 |
| GO:0006006 | glucose metabolic process | 12 | 5.83E-05 |
| GO:0044281 | small molecule metabolic process | 48 | 1.08E-04 |
| GO:0006096 | glycolysis | 6 | 1.19E-04 |
| GO:0006007 | glucose catabolic process | 6 | 2.97E-04 |
| GO:0044282 | small molecule catabolic process | 13 | 3.01E-04 |
| GO:0042692 | muscle cell differentiation | 15 | 3.50E-04 |

Note: GO analysis is based on the Biological Process categories
